# Retraction ‘Reduced miR-125a-5p level in non-small-cell lung cancer is associated with tumour progression’

**DOI:** 10.1098/rsob.200205

**Published:** 2020-07-22

**Authors:** Open Biology Editorial Team

*Open Biol.* **8**, 180118. (Published 10 October 2018). (doi:10.1098/rsob.180118)

The *Editor-in-Chief* in agreement with the Publisher has retracted this article. Following an investigation, we have found that the published manuscript appears to contain substantial overlap with a previously published manuscript [1]. This paper has been identified among of a set of papers that present significant text and data duplication and therefore the data reported in this article are unreliable. The authors and the institution have not responded to any correspondence about this retraction.

Prof. Jon Pines FRS *Editor-in-Chief, Open Biology*
